# Preconception Care Education for Women With Diabetes: A Systematic Review of Conventional and Digital Health Interventions

**DOI:** 10.2196/jmir.5615

**Published:** 2016-11-08

**Authors:** Chidiebere Hope Nwolise, Nicola Carey, Jill Shawe

**Affiliations:** ^1^ School of Health Sciences Faculty of Health & Medical Sciences University of Surrey Guildford United Kingdom

**Keywords:** preconception care, education, diabetes mellitus, women, review, smartphone, mobile applications, technology

## Abstract

**Background:**

Worldwide, 199.5 million women have diabetes mellitus (DM). Preconception care (PCC) education starting from adolescence has been recommended as an effective strategy for safeguarding maternal and child health. However, traditional preconception care advice provided by health care professionals (HCPs) within clinic settings is hindered by inadequate resources, suboptimal coverage, and busy clinics. Electronic health (eHealth), which is instrumental in solving problems around scarce health resources, could be of value in overcoming these limitations and be used to improve preconception care and pregnancy outcomes for women with DM.

**Objective:**

The objectives were to: (1) identify, summarize, and critically appraise the current methods of providing PCC education; (2) examine the relationship between PCC educational interventions (including use of technology as an intervention medium) on patient and behavioral outcomes; and (3) highlight limitations of current interventions and make recommendations for development of eHealth in this field.

**Methods:**

Electronic databases were searched using predefined search terms for PCC education in women with type 1 or 2 DM for quantitative studies from 2003 until June 2016. Of the 1969 titles identified, 20 full papers were retrieved and 12 papers were included in this review.

**Results:**

The reviewed studies consistently reported that women receiving educational interventions via health care professionals and eHealth had significantly improved levels of glycosylated hemoglobin (*P*<.001) with fewer preterm deliveries *(P*=.02) and adverse fetal outcomes (*P*=.03). Significant improvements in knowledge (*P*<.001) and attitudes toward seeking PCC (*P*=.003) were reported along with reduced barriers (*P*<.001).

**Conclusions:**

PCC has a positive effect on pregnancy outcomes for women with DM. However, uptake of PCC is low and the use of eHealth applications for PCC of women with DM is still in its infancy. Initial results are promising; however, future research incorporating mobile phones and apps is needed. Clearly, there is much to be done if the full potential of eHealth PCC to improve obstetric outcomes for women with DM is to be realized.

## Introduction

Electronic health (eHealth) is transforming health care delivery [1-9] and increasingly being used to promote healthy behaviors in people with diabetes mellitus (DM) [10-17]. eHealth is the cost-effective and secure use of information and communication technologies (ICT) in support of health and health-related fields, including health education, knowledge, and research [1]. eHealth plays an instrumental role in improving access to health care, particularly where resources are scarce, and encourages individuals to actively connect with health care services [6,18]. eHealth technologies include consumer health informatics, the Internet, and mobile devices [19]. The Internet has emerged as a popular source of health care information that may replace face-to-face consultations, strengthen patient participation, and supplement health care [20].

A recent report on Internet use [21] identified that of the average 5.6 hours spent on the Internet per day, 51% of time was spent accessing it via mobile devices compared with computers or laptops (42%) and other connected devices (7%). By providing individuals with increased access to information anytime and anywhere, eHealth delivered via mobile phones has significant potential to transform health care delivery. Evidence suggests that 90% of the world’s population own a mobile phone, and over a third of the 7.1 billion mobile devices in use are now smartphones [22,4,23-26] that run third-party apps. Apps are programs designed to enhance smartphone functionality and their increased popularity has resulted in proliferation of educational, decision support, and patient monitoring apps [24]. In 2010, over 200 million health apps were downloaded with estimates suggesting that this figure will have risen to 1.7 billion by 2017 [22].

eHealth technologies can be used to maximize preventative health care for people with chronic conditions such as DM. Worldwide 415 million people have DM, of which 199.5 million are women [27]. DM is now of increasing concern in the field of women’s health and the most common preexisting medical condition complicating pregnancy [28,29]. Poorly-controlled DM at conception coupled with unplanned pregnancy is a major contributor to morbidity and mortality including miscarriages, maternal and perinatal death, and congenital malformations [29,30-36]. It is therefore recommended that women optimize their health via preconception care (PCC) [29,36-42]. Women are also encouraged to achieve a target glycosylated level of hemoglobin (HbA1c; average blood glucose level over the past 2-3 months) of <7% before and during the first trimester of pregnancy to reduce obstetric risks [29,36,38-41]. However, less than 50% of women with DM receive PCC advice [34,43,44] with fragmented and suboptimal services being reported [45-47]. As a result, women with DM have insufficient knowledge of the risks associated with pregnancy to themselves or their baby [12,48,49]. International clinical guidelines [29,38-41] recommend PCC education from adolescence for all women with DM as an effective strategy to facilitate behavior change and improve pregnancy outcomes. However, barriers such as inadequate resources, busy clinics, time, and distance to health facilities [48,50] can inhibit and restrict the extent to which women engage in PCC. Hence, eHealth could be of value in overcoming these limitations and extending the reach of health interventions.

While rapid advances in eHealth technology create a new opportunity to improve knowledge and health outcomes, to date there is no extant literature appraising and quantifying the impact of different methods of PCC provision for women with DM. Therefore, a systematic literature review was undertaken to (1) identify, summarize, and critically appraise the current methods of providing PCC education; (2) examine the relationship between PCC educational interventions (including use of technology as an intervention medium) on patient and behavioral outcomes; and (3) highlight limitations of current practice and make recommendations of eHealth in this field.

## Methods

### Search Strategy

A systematic approach was used to search the literature for relevant articles. The review was limited to human studies conducted between 2003 and June 2016 to reflect current and emerging trends in design and conduct of PCC interventions for women with DM. The reviewed literature drew on a wide range of evidence. The following databases were searched: Medline, Embase, Web of Science, Maternity and Infant Care, Cumulative Index to Nursing and Allied Health, CAB Abstract, British Nursing Index, PsycINFO, Scopus, Science Direct, and Google Scholar.

The keywords “preconception care,” “education,” “counseling,” “diabetes,” “pregnancy outcomes,” “knowledge,” “behavior change,” “birth defects,” and “women” were used in various combinations when searching the databases (see Multimedia Appendix 1 for full text of search string). Additionally, reference lists of retrieved articles, reviews, and related articles were hand-searched for potentially relevant papers. Emphasis was placed on primary research. No language restriction was applied to the search.

### Study Selection

The titles, abstracts, and full papers were screened by CHN and checked by NC and JS. Articles were excluded if there was an agreement that the article met 1 or more of the following exclusion criteria: did not contain any human data; contained no original data (ie, was a commentary, meeting abstract, or editorial); population of interest was not women with DM; and did not assess impact of a PCC educational intervention. The search protocol included identification of potentially relevant articles, screening of identified papers based on their titles and abstracts, examination of full text of potentially relevant studies for eligibility, and application of the inclusion criteria to select the studies included in the review. For the study to be included in the literature review, the following inclusion criteria were applied.

- Women of reproductive age with preexisting type 1 diabetes mellitus (T1DM) or type 2 diabetes mellitus (T2DM) not pregnant at the time of the PCC intervention.
- PCC interventions including but not limited to education, advice, or counseling on use of folic acid, insulin therapy, glycemic control, screening for diabetes complications, contraception use, and blood glucose monitoring.
- Comparator was standard care in all studies except the one [12] in which the intervention group also served as the control.
- Studies reporting maternal and neonatal outcomes and knowledge and attitudes toward PCC.
- Quantitative studies, that is randomized controlled trials, before and after studies, and observational (cohort, cross-sectional and case control) studies.

### Data Abstraction

The data was subsequently extracted by CHN and checked by NC and JS for accuracy and completeness. The reviewers were not masked to the articles’ authors, journals, or institutions.

### Quality Assessment

Assessment was initially performed by CHN and results agreed by NC and JS. The quality of reviewed studies was assessed using a modified version of the EPHPP quality assessment tool for quantitative studies which was developed by the Effective Public Health Practice Project (EPHPP), Canada. It contains summary judgments and an accompanying dictionary that increases standardization of the study quality assessment [51,52]. This tool includes items on selection bias, study design, confounders, blinding, data collection, and withdrawals and dropouts. Each of these 6 aspects of quality received a score out of 3 to make up a total score of 18. The studies were given a rating out of 18, and the quality of the evidence was graded as strong (rating> 14), moderate (rating 7-13), or weak (rating 1-6).

### Synthesis

Meta-analysis of the data was not appropriate because there was great diversity in the interventions, research designs (methodology), and outcome measures. In this review, the main focus was on extracting data on descriptions of interventions (study design, samples, and intervention overviews), outcome measures, and examinations of the effectiveness of interventions. The results are presented as a narrative summary.

## Results

### Search Results

**Figure 1:**
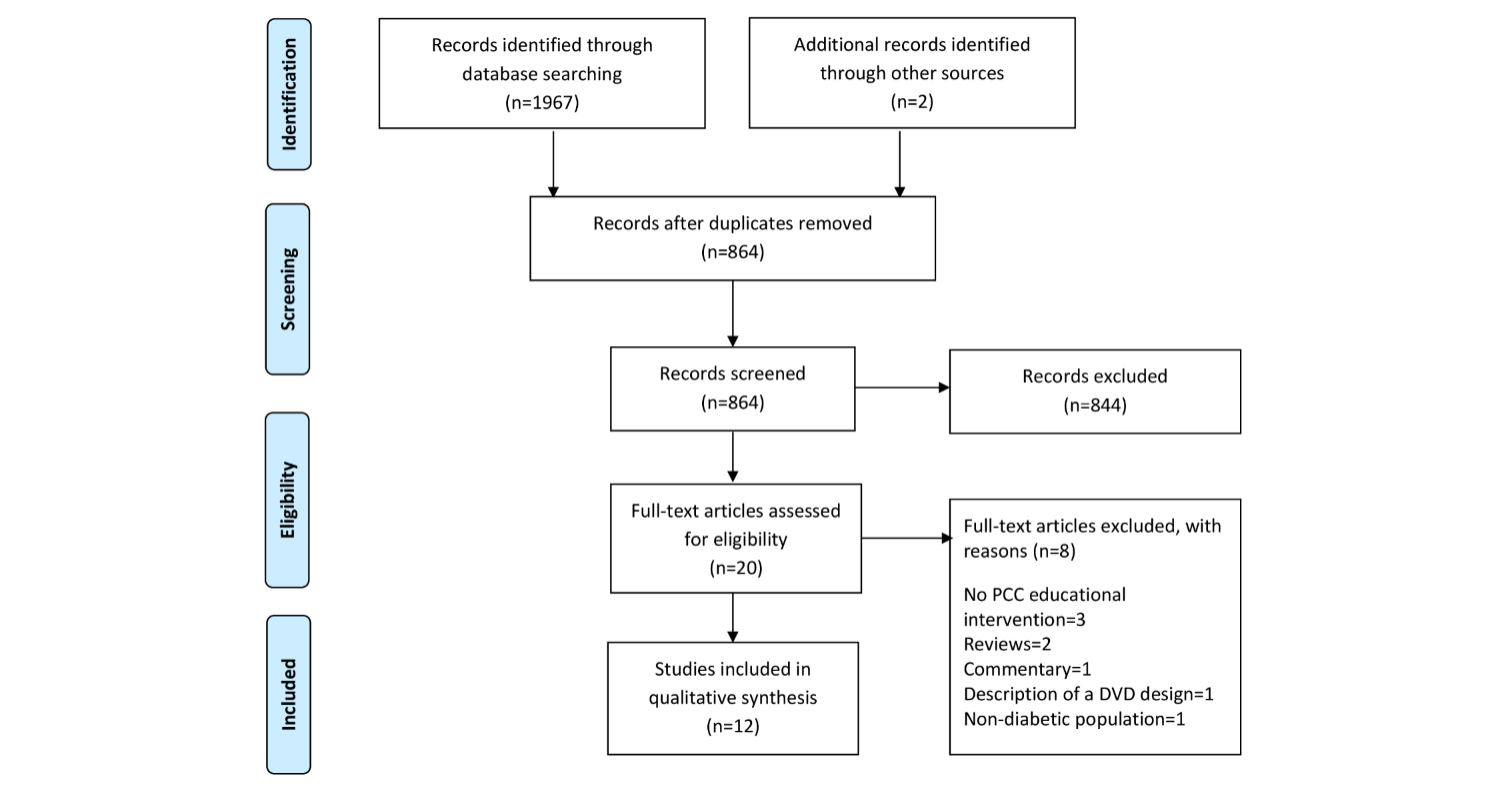
Preferred reporting items for systematic reviews and meta-analyses (PRISMA) flowchart of included studies. PCC: preconception care.

A total of 1969 articles were identified from the literature search and the titles and abstracts of 864 articles were screened for eligibility. After excluding 844 articles that did not meet the eligibility criteria, 20 full text articles were selected for detailed review, of which 12 met the eligibility criteria (Figure 1).

The 12 included studies evaluated 2 categories of PCC health education delivery in use for women with DM: health education provided by health care professionals (HCPs; n=8) and health education using eHealth technologies (CD-ROMs and DVDs; n=4). Of the included studies, 8 were found to investigate the effect of PCC education on maternal and child health outcomes, whereas 4 focused on use of eHealth technology for PCC of women with DM. Of the 12 included articles, 1 study discussed their findings in 2 articles [53,54]. Hence, 12 articles of 11 studies were included.

### Study Characteristics

The summary characteristics of reviewed articles are given in Multimedia Appendix 2. All studies provided face-to-face or eHealth PCC education to women with DM. Women were recruited from specialist and primary care diabetes clinics. Of the included studies, 8 focused on the effect of a PCC intervention on maternal and child outcomes [43,44,53-58] and 4 on improving knowledge and changing attitudes toward PCC [10,11,12,13]. Timing and duration of intervention for some studies was not specified [12,44,55-58]. Follow-up periods ranged from 3 months to 12 years.

All studies were carried out in clinical settings, except one [12], undertaken in women’s homes. Most of the studies were observational [43,44,53-58], with data collected from medical, pregnancy and birth records, or databases. Of the included studies, 4 [10-13] used previously validated and reliable questionnaires. Although data collection methods were different for the face-to-face and eHealth PCC studies, there was consistency in findings and methods of data collection used within each category. Sample sizes ranged from n=58 to n=680. All studies, except one [12], had a separate intervention and control group. All studies were carried out in developed country settings (United States, n=3; United Kingdom, n=5; France, n=1; Spain, n=1; Finland, n=1; and Republic of Ireland, n=1), highlighting increased prioritization of PCC for women with DM in these countries. Studies which adopted eHealth for PCC of women with DM were based in either United States (n=3) [10,11,13] or United Kingdom (n=1) [12], perhaps reflecting the increasing use of ICT to support PCC service provision in these countries.

### Study Quality

Studies varied with respect to their quality (See Multimedia Appendix 2). Of the included studies, 3 had a rating above 14 [11,13,43] and 9 were rated between 7-13 [10,12,44,53-58]. All studies used appropriate study designs, namely, randomized controlled trials, before and after, and cohort studies but lacked details on blinding and allocation concealment. Although small sample sizes [10,11,12], selection bias [10,12,58], and confounding [12,56,57] were underlying issues of weakness within most studies, these were acknowledged and addressed by the authors.

### Findings

Of the included articles, 12 of them reporting on 11 studies (n=12) were grouped into 2 main categories based on their mode of PCC health education delivery: (1) evaluation of PCC education provided by HCPs (n=8) and (2) evaluation of PCC education provided via eHealth technology (n=4).

### Evaluation of PCC Education Provided by HCPs

PCC education traditionally provided in clinical settings by health care professionals is associated with positive maternal and child health outcomes. An overview of the interventions, outcome measures, and their effects are described in the following points.

#### Maternal Health Outcomes

Of the included studies, 2 [56,57] explored the effect of a PCC educational intervention on levels of glycosylated hemoglobin (HbA1c). Boulot et al [56] assigned women with T1DM and T2DM to either an intervention group (n=175) where they received the PCC education before conception, or to a control group (n=360) receiving standard care. Results showed that the educational intervention was effective in enabling more women in the intervention group attain HbA1c <8%. Intervention participants had improved HbA1c in the first trimester with a significantly lower number of women with T1DM (4.3% vs 55%) and T2DM (2.9% vs 27.9%) having HbA1c >8% compared with those in the control group (*P*<.001). A similar study by Galindo et al [57] in Spain included women with both T1DM and T2DM. The intervention group (n=15) received preconception counseling, whereas the control group (n=112) only presented to medical care when pregnant. Although Galindo et al [57] did not set out to measure the effect of a PCC intervention on maternal HbA1c, the intervention group had significantly improved HbA1c (<7%) compared with those in the control group (*P*=.02).

Another UK study [53,54] considered the effect of PCC education on maternal HbA1c, spontaneous abortion, preterm deliveries, and gestational age at presentation for prenatal care. Statistically significant differences were found in intervention participants who had improved and sustained HbA1c (6.5% vs 7.6%; *P*<.001) throughout pregnancy, presented earlier for prenatal care (6.6 vs 8.3 weeks; *P*<.001), less spontaneous abortions (*P*=.06), and preterm deliveries (*P*=.02).

Furthermore, 2 other studies [43,44] reported the effects of PCC education on HbA1c, gestational age at presentation for prenatal care, and folic acid intake in women with T1DM and T2DM. During the 3-year study period by Murphy et al [43], women who received a structured education program were assigned to the intervention group (n=181) and those who did not, to a control group (n=499). Women in the intervention group with increased intake of 5mg folic acid before conception (*P*<.001) had significantly improved HbA1c values (6.9% vs 7.6%; *P*<.001), and an earlier date of presentation for prenatal care compared with those in the control group (6.7 vs 7.7 weeks; *P*<.001). The role of PCC education in promoting healthy preconception behaviors and pregnancy planning was also explored by Tripathi et al [44] who assigned women receiving PCC counseling to the intervention group (n=240) and those who did not, to the control group (n=297). Results showed that participants receiving the intervention had significantly improved and sustained levels of HbA1c (≤7% vs >7%) 3 months before conception (*P*=.002) and during the first trimester of pregnancy (*P*<.001), higher rates of folic acid intake 3 months before pregnancy (*P*<.001), and presented earlier for prenatal care (≤8 weeks vs >8 weeks; *P*=.001).

Additionally, 2 recent studies [55,58] reinforced the benefits of PCC education on HbA1c and pregnancy outcomes. Neff et al [55] assigned women with T1DM to the intervention group where they received health education (n=70) while those in the control group received standard care (n=394). Intervention participants had significant improvements to HbA1c <7% (6.9% vs 7.8%; *P*<.001) and earlier prenatal care presentation (6±2 weeks vs 8±6 weeks; *P*<.001) compared with those who received standard care. However, the effect on rates of spontaneous abortion or preterm delivery was not found to be statistically significant (*P*=.12, *P*=.46 respectively). Kekalainen et al [58] also found statistically significant differences in the intervention group who had improved and sustained HbA1c (7.1% vs 9.1%; *P*<.001) and reduced adverse pregnancy outcomes (*P*=.06).

#### Child Health Outcomes

Boulot et al [56] demonstrated that women with T1DM who received PCC education had significantly lower rates of perinatal mortality and congenital malformation (*P*<.005) compared to those in the control group. Furthermore, women with DM whose HbA1c was > 8% in the first trimester had double the risk of developing adverse fetal outcomes such as perinatal mortality (*P*<.005), congenital malformation (*P*<.01), and preterm delivery (*P*<.005).

Additionally, 5 further studies [43,53,54,57,58] reported similar findings. Temple et al [53,54] found that women who received a PCC educational intervention had significantly reduced risk of adverse outcomes (including malformations, stillbirths, and neonatal death) compared with those receiving standard care (*P*=.03). Similarly, Murphy et al [43] and Kekalainen et al [58] found that the intervention group participants experienced a significant reduction in congenital malformations compared with those in the control group (*P*=.009; *P*=.001). Galindo et al [57] also found a positive relationship between increase in maternal HbA1c levels (>7%) and the occurrence of fetal malformations. Additionally, Tripathi et al [44] and Neff et al [55] found a significant association between lack of preconception care education and increased risk of adverse fetal outcomes (*P*=.03).

Most studies (n=7) reported low levels of PCC uptake, range 12% [57] to 48.5% [56], amongst women with DM.

### Evaluation of PCC Education Provided via eHealth Technology

Low levels of PCC uptake among women with DM have elicited interest in use of multimedia technologies such as CD-ROMs and DVDs as an intervention tool for PCC education.

Four studies [10-13] investigated the effect of eHealth technology on knowledge and PCC behaviors. Charron-Prochownik et al [10] developed and used an interactive computer program (CD-ROM) to promote PCC knowledge. Adolescent girls with T1DM were randomized to receive the 3-month CD-ROM intervention (n=37) or standard care (n=16). Significant improvement in knowledge (*P*<.05), perceived benefits (*P*=.04), and reduced barriers to seeking PCC (*P*=.01) were reported in intervention participants. An RCT by Fischl et al [11], which lasted 9 months, similarly used an interactive CD-ROM to deliver PCC health education. Adolescent girls with T1DM were randomized to either the intervention group (n=43) where they watched 2 CD-ROMs, read a book, and met with a nurse for counseling or standard care (n=45). Compared with those receiving standard care, intervention participants had significantly improved knowledge and perceived benefits of PCC (*P*<.001), reduced barriers to seeking PCC (*P*<.001), and increased intention to initiate PCC discussion with health care professionals (*P*<.001). The effect on intention to use contraception was not significant (*P*=.10).

A UK study by Holmes et al [12] aimed to explore whether an educational DVD would improve PCC knowledge and behavior. Women with T1DM and T2DM (n=97) who viewed the contents of the DVD individually in their homes showed a significant increase in perceived benefits and attitudes to contraceptive use (*P*=.001), receiving PCC (*P*=.003), knowledge of pregnancy planning (*P*<.001), and pregnancy-related risks (*P*<.001). Finally, Charron-Prochownik et al [13] assessed the long-term effect (12 months) of an educational DVD on knowledge and attitudes to PCC in adolescent girls with T1DM and T2DM. Participants who were randomized to receive the intervention (n=51) demonstrated a significant increase in PCC knowledge (*P*=.001), and intention to discuss PCC and contraception with health care professionals (*P*=.03, *P*=.003), compared with those in the control group who received standard care (n=58).

## Discussion

### Principal Findings

The reviewed evidence suggests that educationally-based PCC (delivered by health care professionals) is effective in improving maternal and child health. The evidence is consistent across studies, but with few robust controlled studies of PCC educational interventions for women with DM. Studies are generally of moderate quality, with only one assessed as high quality [43]. The inadequacy in traditional PCC education in meeting the needs of women with DM has been widely recognized [43,44,48,56-58], but alternative means of providing PCC remains underresearched. This review highlights the potential capacity of eHealth technologies to help improve coverage and access to PCC.

PCC should ideally be provided to all women with DM [29,34]. However, evidence presented in this review confirms that PCC uptake is still <50% [44,55-57], in line with the low PCC uptake reported in the 2007 confidential enquiry into maternal and child health (CEMACH) in women with DM [34]. Women who do not receive PCC also have poor levels of glycemic control, higher rates of unplanned pregnancy, and adverse pregnancy outcomes [29,30,43,53,54,56-58]. It is therefore worrying that PCC service provision and uptake has not increased at the same rate as the prevalence of DM in women of reproductive age. PCC provided predominantly in a health care setting by a HCP also excludes the 55% (3.1 billion) of the developing world’s population in rural areas who do not have adequate access to health care [59]. PCC provision is therefore almost nonexistent for many women in the developing world who have increased risk of adverse maternal and fetal outcomes [37]. This underlines the shortcoming of traditional PCC practice. We have reached the age of personalized medicine [23]. The growing popularity and effectiveness of eHealth technologies for health promotion in several areas including obesity and smoking cessation [4,15-17,60-62], makes its use in PCC of women with DM timely, warranting further exploration.

eHealth technologies hold great promise in terms of helping to deliver preconception health education that increases knowledge and supports behavior change [10-13]. This review highlights the potential of these technologies to empower women with DM to make informed reproductive health decisions. The ultimate goal is to prevent unplanned pregnancies and reduce adverse maternal and fetal outcomes. Behavioral interventions must reach the target population to achieve success [62]; in this lies a weakness of the reviewed eHealth intervention studies which have used technology that is now dated and offers limited scope to the many women who do not have access to computers and/or DVD players [10-13].

### Challenges of eHealth PCC

This review highlights that adoption of eHealth in this field is slow and use of ICT for PCC is still very limited. For example, between 2008 and 2016, only 4 studies examined the effect of eHealth PCC using multimedia technology—CD-ROMs and DVDs, with none examining the use of the Internet or mobile phones. Computers or ICT has been used by some reviewed studies to provide health education within clinic settings [10,11,13]. However, people are now proactive in seeking health information and increasingly prefer to do things in the privacy of their homes and in their own time. Developments in technology mean that increasingly health programs can be delivered to people outside the traditional clinic setting, improving access for hard to reach populations across the world, as reflected in the recently agreed goals of the United Nations sustainable development plan [63].

The majority of studies (n=11) involved women traveling to clinics to physically receive the PCC educational intervention. However, constraints such as inadequate resources, time, and distance to health facilities have been shown to inhibit women’s ability to adequately access such PCC interventions [48,50], and for many women around the world, this has negative implications for PCC uptake. Furthermore, no studies were carried out in developing countries; reflecting the existent inequality in PCC service provision. Mobile technologies can be used to extend the reach of PCC interventions given that 90% of the world’s population now have access to a mobile phone [25]. Moreover, evidence of a reverse digital divide confirms that low income populations and those living in resource-poor settings are among the fastest growing users of mobile phones [64,65].

Bull [64] argues that if more people can be reached with health promotion interventions then even “modest” effects will translate into greater impact on morbidity and mortality. Contemporary eHealth technologies have the capacity to take an intervention that works on a small scale to a larger audience. From this review, which demonstrates the efficacy of PCC health education, it is apparent that the challenge lies in translating “what works” to a wider audience. We have a unique opportunity to overcome this challenge in eHealth PCC using mobile phones.

### Way Forward for eHealth PCC

Mobile phones represent an underutilized resource that could be developed to support eHealth interventions for women with DM. Mobile phone ownership in developed countries has outstripped the population, with an average phone ownership of 1.16 mobile phones per person [25]. In developing countries, mobile communications technology is the fastest growing sector of the telecommunications industry with over a billion mobile phones [65,66]. Smartphones in particular, have the capacity of both computers and the Internet [24]. Their significant advantage over desktop computers, laptops, and DVD players make them a valuable tool for giving more women access to PCC [46]. They offer the opportunity to penetrate a larger population, are easily accessible, technologically advanced, utilize existing features (eg, geo-positioning technology; Internet access with photos, videos, and voice-recording capabilities), are mobile and convenient to use [4,22,23,67].

Many of the advanced functionalities of smartphones are aided by software applications or apps which hold great potential in helping to deliver cost-effective health interventions [4,16,17,22,23,67]. 90% of the time spent on mobile phones is spent on apps and in terms of usability, they are preferred over Web or computer-based applications [14,68]. Incorporating health education interventions into apps could help reduce barriers to adoption and facilitate increased acceptance of the intervention [4,24,69]. The innovative integration of smartphones or apps and PCC health education could help reduce the widespread burden caused by unplanned pregnancies in women with DM.

This is the first review to incorporate the use of eHealth technologies for PCC of women with DM into a discussion of PCC interventions. It highlights the benefits and limitations of each mode of delivery, and recommends use of smartphones and apps for maximizing the impact of future PCC interventions.

### Limitations

A number of limitations should be noted. All reviewed studies were conducted in developed countries and their generalizability is limited to the geographical locations and health care settings in which the studies have been conducted. Various research methodologies were used in this review, and study quality was mainly moderate. Methodological weaknesses present in the study designs (small sample sizes, selection bias, confounding, and short follow-up periods) require caution in interpreting the results.

### Conclusions

PCC education has a positive effect on pregnancy outcomes for women with DM. However, uptake of PCC is low and the use of eHealth apps for PCC of women with DM is still in its infancy. eHealth apps have the potential to improve access to PCC around the world, particularly in developing countries where women have increased risk of adverse maternal and fetal outcomes. Further research utilizing smartphones and apps is urgently needed as these technologies are increasingly being used around the world to provide health care information and support. Clearly, there is much to be done if full potential of eHealth PCC to improve obstetric outcomes for women with DM is to be realized.

## Appendix

Multimedia Appendix 1 Medline Strategy.

Multimedia Appendix 2 Summary characteristics and quality assessment of reviewed articles.

## Abbreviations

ACE: angiotensin-converting enzyme
CEMACH: confidential enquiry into maternal and child health
DM: diabetes mellitus
DSN: Diabetes specialist nurse
HbA1c: glycosylated haemoglobin
HCPs: Health care professionals
ICT: Information and communication technology
PCC: preconception care
PNC: prenatal care
RCT: randomized controlled trial
READY-Girls: reproductive-health education and awareness of diabetes in youth for girls
T1DM: type 1 diabetes mellitus
T2DM: type 2 diabetes mellitus
