# Supplementary material for: Preconception Care Education for Women With Diabetes: A Systematic Review of Conventional and Digital Health Interventions
Source: J Med Internet Res. 2016 Nov 8;18(11):e291. doi: 10.2196/jmir.5615 (PMC5120238; doi:10.2196/jmir.5615)
Supplement: Multimedia Appendix 1 [file jmir_v18i11e291_app1.pdf]

APPENDIX 1: Detailed Electronic Database Search Strategies

Medline Strategy

Terms	Returns
(pre#concept* care OR pre#concept* OR pre#concept* counse*ing OR pre#concept* education OR pre#pregnan* care OR pre#pregnan* plan* OR pre#pregnan* OR pre#concep* adj1 care) AND (diabetes OR diabetes mellitus OR diabet* OR type 1 diabetes OR type 2 diabetes OR insulin dependent diabet* OR non#insulin dependent diabet* OR IDDM OR NIDDM) AND (anomal* OR congenital anomal* OR malformation* OR malformations* OR birth defect* OR health outcome* OR pregnancy outcome* OR obstetric outcome* OR adverse adj1 outcome* OR knowledge OR attitude OR behaviour change) AND (maternal OR maternal health OR mother* OR gestation OR pregnan* OR wom*n OR infant OR f*tus OR neonat* OR newborn* OR child).	375
