# Supplementary material for: Preconception Care Education for Women With Diabetes: A Systematic Review of Conventional and Digital Health Interventions
Source: J Med Internet Res. 2016 Nov 8;18(11):e291. doi: 10.2196/jmir.5615 (PMC5120238; doi:10.2196/jmir.5615)
Supplement: Multimedia Appendix 2 [file jmir_v18i11e291_app2.pdf]

Table 1. Summary characteristics and quality assessment of reviewed articles

Author or country	Study population	Methods or focus	Intervention	Findings	Quality assessm ent
Boulot et al [56] or France	435 pregnant women with DM[a].<br><br>T1DM[b], n=289 (intervention =140/289, 48.5% received the intervention, control=149).<br><br>T2DM[c], n=146 (intervention= 35/146, 24% received the intervention, control=111).	Prospective cohort study.<br><br>To determine if PCC[d] interventions improved pregnancy outcomes in women with DM.	Oral PCC educational intervention delivered by health care professionals (HCPs[e]; diabetologists).<br><br>Advice regarding blood glucose optimization, diabetes complications, dietary modification, self-monitoring of blood glucose levels, and insulin therapy.<br><br>Information was collected on HbA1c[i]>8% in the first trimester.<br><br>The duration of intervention was not specified.<br><br>Face-to-face time with the women was not indicated.	Significantly lower no of women with HbA1c >8% in intervention group compared with control (4.3% vs 55%; *P*<.001 and 2.9% vs 27.9%; *P*<.001) for T1DM and T2DM, respectively).	(9/18)
Galindo et al [57] or Spain	127 Women with T1DM and T2DM.<br><br>Intervention, n=15/127, 12% received the intervention, control, n=112.	Prospective cohort study.<br><br>To investigate the effect of DM on pregnancy outcome.	Oral PCC educational intervention delivered by health care professionals (HCPs).<br><br>Advice regarding self-monitoring of blood glucose levels and intensification of insulin therapy.<br><br>Duration of intervention was not specified.<br><br>Face-to-face time with the women was not indicated.	3/15 of pregnancies in intervention group and 14/112 in control group resulted in congenital malformations.<br><br>1 spontaneous abortion was recorded in the intervention group compared with 9 in the control.	(9/18)
Temple et al 2006 [53, 54] or United Kingdom	290 pregnancies to women with T1DM.<br><br>Intervention, n=110/290, 37.9% received the intervention, control, n=180.	Prospective cohort study.<br><br>To investigate the relationship between PCC and obstetric outcomes in women with T1DM.	Oral PCC educational intervention provided by HCPs (doctors, dieticians and DSNs[f]).<br><br>Advice regarding blood glucose monitoring, glycemic control, and initiation of folic acid supplements after stopping contraception, smoking cessation, and avoidance of teratogens such as ACE[g] inhibitors and statins.<br><br>Targets were set for pre and postprandial blood glucose levels. The intervention group attended a PCC center at intervals of 1-3 months.	There was lower spontaneous abortion (5.7% vs 14.0%; *P*=.06), adverse pregnancy outcome (2.9 vs 10.2; *P*=.03), and premature delivery (5.0% vs 14.2%; *P*=.02) rates in the intervention group compared with control.	(13/18)
Author or	Study	Methods or	Intervention	Findings	Quality

country	population	focus			assessment
Tripathi et al [44] or United Kingdom	588 women with T1DM and T2DM.<br><br>Intervention, n=240/588, 40.8% received the intervention, control, n=297.	Prospective cohort study.<br><br>To investigate the association between PCC and obstetric outcomes, as well as pregnancy planning indicators.	Oral PCC educational intervention provided by HCPs.<br><br>Counseling regarding PCC.<br><br>The content of the intervention was not described.<br><br>Duration of the intervention was not specified.<br><br>Face-to-face time with women was not indicated.	PCC intervention led to significant improvements in HbA1c before (OR[j] 1.91, 95% CI 1.10 to 3.04; *P* =.002) and during pregnancy (OR 2.05, 95% CI 1.39 to 3.03); *P*< .001), and in folic acid intake (OR 4.88, 95% CI 3.26 to 7.30; *P* <.001) in intervention group compared with control.	(11/18)
Murphy et al [43] or United Kingdom	680 women with T1DM and T2DM.<br><br>Intervention, n=181/680, 26.6% received the intervention, control, n=499.	Prospective cohort study.<br><br>To investigate the association between PCC and obstetric outcomes.	Oral PCC educational intervention delivered by HCPs (doctors, nurses, and midwives).<br><br>Structured education regarding PCC.<br><br>The content of the intervention was not described in the paper.<br><br>Frequency of face-to-face time with the women ranged from 1 to 7.	Intervention group presented significantly earlier for PNC[h] (6.7 vs 7.7 weeks; *P* <.001), and was unlikely to be taking ACE inhibitors (1.1 vs 4.6%; *P* =.05) and statins (0 vs 7.6%; *P*<.001) at conception compared with control.<br>HbA1c improved in the intervention group and was sustained through the first trimester (6.9% vs 7.6%; *P*<.001).	(14/18)
Neff et al [55] or Republic of Ireland	505 women with T1DM.<br><br>Intervention, n= 70/505, 14% received the intervention, control, n=394.	Retrospective cohort study.<br><br>To analyze effect of PCC on obstetric outcomes.	Oral educational intervention provided by HCPs (DSNs, endocrinologists, dieticians).<br><br>Advice regarding insulin therapy, discontinuation and replacement of teratogens such as ACE inhibitors and statins, intake of 5mg folic acid and retinal assessment.<br><br>Face-to-face time with women was not indicated.	PCC education led to better HbA1c (6.9 vs 7.8%); *P* <.001 and earlier presentation for PNC [(6 ± 2 vs 8 ± 6 weeks; *P* <.001) in the intervention group compared with control.	(13/18)
Kekalainen et al [58] or Finland	145 pregnancies to women with T1DM.<br><br>Intervention, n=96/145, 66% control, n=49.	Retrospective cohort study.<br><br>To evaluate the effect of pregnancy planning on glycemic control and pregnancy outcomes.	Oral educational intervention provided by HCPs (doctors).<br><br>Advice regarding glycemic control, screening for diabetes complications and hypertension, medication review, and folic acid supplementation.<br><br>Face-to-face time with women not indicated.	Intervention group had significantly lower HbA1c before conception (7.06% vs 9.11%; *P* <.001) which was sustained throughout pregnancy (6.37% vs 7.28%; *P* <.001). Congenital malformation rate was lower (2.5% vs 11.1%; *P* <.001) in the intervention compared with control.	(12/18)
Author or country	Study	Methods or	Intervention	Findings	Quality

	population	focus			assessment
Charron-prochownik et al [10] or United States	53 adolescent girls with T1DM (16-19.9 years).<br><br>Intervention, n=37 (CD-ROM-17, book-20), control, n=16.	Randomized controlled trial.<br><br>To develop and assess the clinical feasibility of a PCC program for young women with DM.	PCC educational intervention provided via eHealth application. READY-Girls program was developed by authors.<br><br>The CD-ROM contained information on DM and its effects on reproductive health, sexuality, puberty and pregnancy; advantages of PCC; sessions on decision making and communication skills development.<br><br>Intervention participants viewed a CD-ROM and received one comprehensive session on the content of the CD-ROM at their routine clinic visit. This lasted about an hour.	Knowledge ($F_{2,40.1}$=3.77; P= .03) and perceived benefits ($F_{2,40.1}$=3.48; P=.04) of PCC significantly improved by the end of the study.<br><br>Significant increase in intention to seek PCC and utilize effective family planning ($F_{1,37=}$ =5.75; P =.02) was recorded.	(12/18)
Fischl et al [11] or United States	88 adolescent girls with T1DM (13-19.9 years).<br><br>Intervention, n=43, control, n=45.	Randomized controlled trial. To assess the effectiveness of PCC on behavioral and cognitive outcomes.	PCC educational intervention provided via eHealth application.<br><br>READY-Girls preconception care program was used to promote the benefit of PCC.<br><br>Intervention participants viewed an educational CD-ROM, read a book, and received a nurse consultation over 3 consecutive clinic visits at intervals of 3 months.	Knowledge (F=32.34; P <.001) and perceived benefit of PCC (F= 9.70; P =.003) increased by the end of the study.<br><br>Significant improvement over time in actual initiation of PCC discussion with diabetes health care team (F= 14.6; P<.001).	(16/18)
Holmes et al [12] or United Kingdom	97 women with DM (16-40 years).<br><br>T1DM, n=89, T2DM, n=8.	Before and after study.<br><br>To determine if an educational DVD increases knowledge and changes attitudes of women toward PCC.	PCC educational intervention provided via eHealth application.<br><br>The authors developed and explored the use of a DVD to raise awareness of pregnancy planning and prevention of unplanned pregnancies.<br><br>Women viewed an educational DVD with information on pregnancy planning, contraception use, pregnancy complications, and PCC advice.<br><br>The number of times they viewed the DVD or the duration of the intervention was not reported.	Significant improvement in self-confidence to use contraception to prevent unplanned pregnancies and access PCC (OR 3.3, CI 1.9, to 4.7; P <.001).<br><br>Significant reduction in perceived barriers to PCC (OR -0.7, CI -1.2 to -0.2); P=.01).	(12/18)
Author or country	Study population	Methods or focus	Intervention	Findings	Quality assessment

Charron-prochownik et al [13] or United States	109 adolescent girls with T1DM and T2DM (13-19 years).<br><br>Intervention, n=51, control, n=58.	Randomized controlled trial.<br><br>To examine the long-term effect of the READY-Girls program on PCC knowledge and behavior.	PCC educational intervention provided via eHealth application.<br><br>READY-Girls PCC program was used to enhance PCC knowledge and behaviors.<br><br>Intervention patients viewed 2 educational DVDs and read a book over 3 consecutive clinic visits at intervals of 3 months.	Increased intention ($F_{6,82.4}$= 2.56; P = .03) to initiate diabetes discussion with HCPs.<br><br>Significant increase in intention to seek PCC and plan a pregnancy ($F_{6,534}$=2.58; P =.02).	16/18

[a]DM: diabetes mellitus.
[b]T1DM: type 1 diabetes mellitus.
[c]T2DM: type 2 diabetes mellitus.
[d]PCC: preconception care.
[e]HCPs: Health care professionals.
[f]DSN: Diabetes specialist nurse.
[g]ACE: angiotensin-converting enzyme.
[h]PNC: prenatal care.
[i]HbA1c: glycosylated haemoglobin.
[j]OR: odds ratio.
[k]READY-Girls: Reproductive-health education and awareness of diabetes in youth for girls.
